# MicroRNA-34a promotes genomic instability by a broad suppression of genome maintenance mechanisms downstream of the oncogene KSHV-vGPCR

**DOI:** 10.18632/oncotarget.7248

**Published:** 2016-02-08

**Authors:** Claudia J. Krause, Oliver Popp, Nanthakumar Thirunarayanan, Gunnar Dittmar, Martin Lipp, Gerd Müller

**Affiliations:** ^1^ Molecular Tumor Genetics and Immunogenetics, Max Delbrück Center for Molecular Medicine in the Helmholtz Association, Berlin, Germany; ^2^ Mass Spectrometry Group, Max Delbrück Center for Molecular Medicine in the Helmholtz Association, Berlin, Germany; ^3^ Laboratory of Molecular Radiology, Center for Disease Biology and Integrative Medicine, Graduate School of Medicine, University of Tokyo, Tokyo, Japan

**Keywords:** KSHV, vGPCR, microRNA-34a, genomic instability, genome maintenance mechanisms

## Abstract

The Kaposi's sarcoma-associated herpesvirus (KSHV)-encoded chemokine receptor vGPCR acts as an oncogene in Kaposi's sarcomagenesis. Until now, the molecular mechanisms by which the vGPCR contributes to tumor development remain incompletely understood. Here, we show that the KSHV-vGPCR contributes to tumor progression through microRNA (miR)-34a-mediated induction of genomic instability. Large-scale analyses on the DNA, gene and protein level of cell lines derived from a mouse model of vGPCR-driven tumorigenesis revealed that a vGPCR–induced upregulation of miR-34a resulted in a broad suppression of genome maintenance genes. A knockdown of either the vGPCR or miR-34a largely restored the expression of these genes and confirmed miR-34a as a downstream effector of the KSHV-vGPCR that compromises genome maintenance mechanisms. This novel, protumorigenic role of miR-34a questions the use of miR-34a mimetics in cancer therapy as they could impair genome stability.

## INTRODUCTION

Kaposi's sarcoma-associated herpesvirus (KSHV), also known as human herpesvirus 8 (HHV-8), is the etiological agent of three associated diseases: Kaposi's sarcoma (KS), primary effusion lymphoma (PEL), and a plasmablastic form of multicentric Castleman disease (MCD). KSHV is a DNA tumor virus that encodes several homologues of cellular genes including the G protein-coupled receptor vGPCR. The receptor shows highest homology to the human chemokine receptors CXCR1 and CXCR2 and is constitutively active in the ligand-unbound state [1]. vGPCR binds a number of human and murine CC and CXC chemokines that modulate its constitutive activity and, when expressed as a transgene in mice, induces KS-like lesions [2].

The presence of viral DNA and proteins can elicit a DNA damage response (DDR) and activate DNA repair pathways in the infected host cell. However, many DNA viruses, including KSHV, are able to subvert the growth-suppressive DDR and downregulate DNA repair mechanisms [3–5]. Infected cells are thus at high risk for an accumulation of genomic aberrations as genome integrity relies on the effectiveness of the DDR and DNA repair mechanisms. Genomic instability has indeed been described in KSHV-infected and primary KS and PEL cancer cells [6, 7], but little is known about the molecular mechanisms by which KSHV induces genomic instability.

MicroRNAs are short, single-stranded RNAs that regulate gene expression on the transcript level [8]. Members of the highly conserved microRNA-34 family have been implicated as tumor suppressors [9, 10] acting downstream of p53, a major effector in the DNA damage response [11]. They are, for example, involved in the control of cellular proliferation, cell cycle, apoptosis and senescence [9, 12]. These findings led to the development of miR-34a mimetics for cancer therapy [13, 14], which currently are in phase I clinical trials [13, 14]. More recent studies, however, suggested that the miR-34 family is dispensable for p53-mediated processes [15] and that high miR-34a levels can be beneficial for certain cancer cells [16–20]. In this connection, Forte and colleagues [21] showed that miR-34a is highly expressed in KSHV-infected lymphoma cell lines, suggesting a potential role of miR-34a in KSHV-dependent tumorigenesis.

Here, we demonstrate that the KSHV-vGPCR impairs the expression of a large number of genes required for the maintenance of genome integrity. A comparison of vGPCR-expressing tumor cell lines that differ significantly in their aggressiveness revealed that this repression is largely mediated by vGPCR-dependent induction of miR-34a, which in turn downregulates these genome maintenance genes. Taken together, our data suggest a novel, oncogenic role for microRNA-34a as it promotes genomic instability and tumorigenesis downstream of the KSHV-vGPCR.

## RESULTS

### vGPCR-expressing tumor cell lines differ in their tumorigenicity

**Figure 1:**
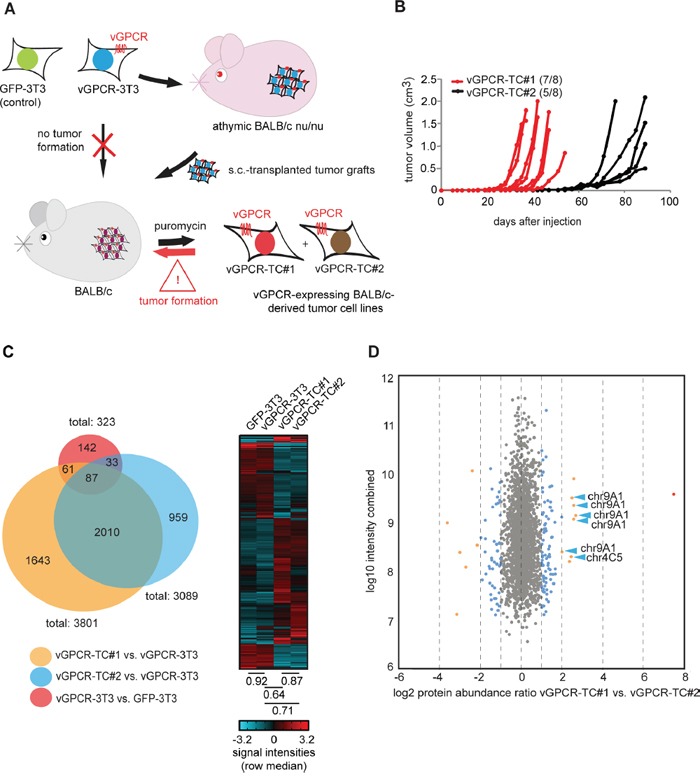
Large-scale analysis of the transcriptome and proteome of cell lines from the vGPCR mouse tumor model **A.** The vGPCR-driven mouse tumor model: vGPCR-transduced BALB/c 3T3 cells (“vGPCR-3T3”) induce tumors in athymic BALB/c nude mice only; however, tumor fragments from nude mice tumors grew progressively in immunocompetent BALB/c mice and gave rise to vGPCR-expressing tumor cell lines (vGPCR-TC#1 and #2) that are capable of directly inducing tumors in BALB/c mice. **B.** Tumor induction and growth rate of vGPCR-TC#1 cells (red) and vGPCR-TC#2 cells (black) in BALB/c mice; each mouse received 1*10^6^ cells s.c., n = 8 per group. **C.** Genome-wide mRNA expression analysis of cell lines derived from the vGPCR-driven tumor model. The Venn diagram indicates the overlap of differentially expressed genes from the following comparisons: vGPCR-TC#1 vs. vGPCR-3T3 (orange), vGPCR-TC#2 vs. vGPCR-3T3 (blue) and vGPCR-3T3 vs. GFP-3T3 (red). The heat map shows a cluster analysis for all genes that are significantly regulated between any two of the following cell lines: GFP-3T3, vGPCR-3T3, vGPCR-TC#1 & #2 (fold-change ≥ 2, p ≤ 0.05). Spearman's rank correlation coefficients of indicated cell lines are depicted below the heat map. **D.** Comparison of relative protein abundances by stable isotope dimethyl labeling (DML): the scatter plot depicts changes in protein abundance between vGPCR-TC#1 and vGPCR-TC#2 cells; DML ratios (log2) are plotted against combined peptide intensities (log10 H+L); chr9A1 and chr4C5/C6-encoded proteins are marked by blue arrows.

We previously described a vGPCR-driven murine tumor model (Figure 1A and ref. [22]) that allows for the identification of crucial mechanisms of cancer development. In this model, BALB/c-3T3 cells were retrovirally transduced with the *vGPCR* oncogene, thus generating a cell line that is tumorigenic in athymic, immunorestricted BALB/c *nu/nu* mice due to the transforming potential of the vGPCR. These “vGPCR-3T3” cells are, however, not tumorigenic in immunocompetent BALB/c mice though fragments from tumors of BALB/c *nu/nu* mice grow progressively when grafted onto BALB/c mice. Strikingly, cell lines generated from tumors of BALB/c mice, named “vGPCR-TC”, are highly tumorigenic in these mice, in contrast to the parental vGPCR-3T3 cell line. Tumor growth of vGPCR-TC cell lines is still dependent on the vGPCR oncogene as shown by shRNA-mediated knockdown of the receptor [22]. A detailed analysis of two independently generated vGPCR-TC cell lines, termed “vGPCR-TC#1” and “vGPCR-TC#2”, revealed a significant difference in their tumorigenicity: 7 out of 8 animals injected with vGPCR-TC#1 cells developed tumors with a median onset (tumor volume > 0.02 cm^3^) of 28 days, whereas only 5 out of 8 animals receiving the replicate cell line vGPCR-TC#2 developed tumors with a median onset of 55 days (Figure 1B). Thus, we were interested in identifying the molecular differences between the two tumor cell lines that enable the more aggressive growth of vGPCR-TC#1 cells.

To this end, we performed a comprehensive, large-scale characterization of the two vGPCR-expressing tumor cell lines, their parental vGPCR-3T3 cell line and a GFP-transduced BALB/c-3T3 (“GFP-3T3”) control cell line. First, we compared the transcriptomes of all cell lines using Affymetrix GeneChip 430 2.0 expression arrays (Figure 1C). Our data showed that the introduction of the vGPCR into BALB/c-3T3 cells had a rather small effect on the overall gene expression pattern as only 323 genes were differentially expressed (≥ 2-fold, p ≤ 0.05) between GFP-3T3 and vGPCR-3T3 cells. In contrast, passaging of vGPCR-3T3 cells through nude and BALB/c mice had a dramatic effect on global gene expression: 3801 genes were regulated between vGPCR-3T3 and vGPCR-TC#1 cells and, likewise, 3089 genes between vGPCR-3T3 and vGPCR-TC#2 cells (Figure 1C). A hierarchical cluster analysis of all differentially expressed genes between GFP-3T3, vGPCR-3T3, vGPCR-TC#1 and vGPCR-TC#2 cells revealed that both tumor cell lines share many similarities but clearly differ from their parental vGPCR-3T3 cell line as well as GFP-3T3 control cells (Figure 1C). We could confirm these results by mass spectrometry-based stable isotope dimethyl labeling (DML) which allowed us to directly compare changes in protein abundance between vGPCR-TC#1 and vGPCR-TC#2 cells and their parental vGPCR-3T3 cell line (Figure 1D and Supplementary Table 1). DML showed that 2066 out of 2206 quantified proteins (∼94 %) are similarly expressed in vGPCR-TC#1 and vGPCR-TC#2 cells (fold-change ≤ 2). Interestingly, we observed a particular high abundance of several chromosome 4- and 9-encoded proteins in vGPCR-TC#1 cells (Figure 1D).

### The aggressively growing cell line vGPCR-TC#1 harbors massive genomic amplifications

**Figure 2:**
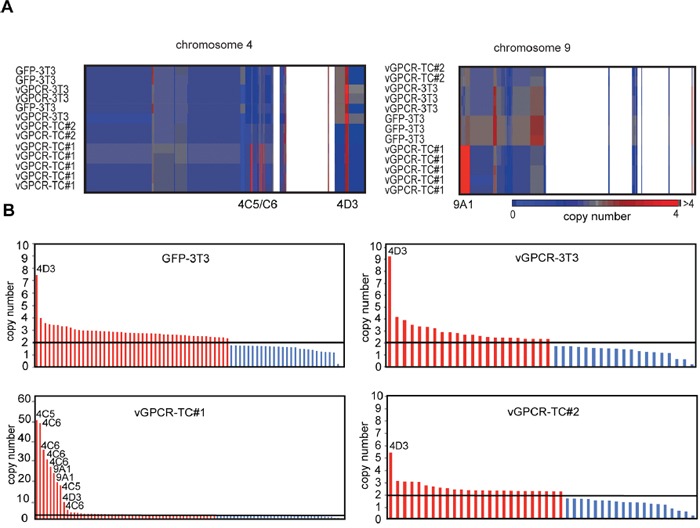
Array-based CGH analysis **A.** Clustered heat map of segments on chromosome 4 and chromosome 9 for the cell lines GFP-3T3, vGPCR-3T3, vGPCR-TC#1 and vGPCR-TC#2 (individual arrays are shown). White areas correspond to regions showing no copy number alteration. **B.** DNA copy number profiles: segments with significant changes in their copy number in GFP-3T3, vGPCR-3T3, vGPCR-TC#1 and vGPCR-TC#2 cells are shown in red (amplifications) or blue (deletions); the diploid status (n = 2) is marked by a black line.

Based on the strong overrepresentation of chromosome 4- and chromosome 9-encoded genes and proteins in vGPCR-TC#1 cells we tested for copy number variations by array-based comparative genomic hybridization (aCGH). aCGH identified major amplifications of the chromosome bands 4qC5, 4qC6 and 9qA1 specifically in vGPCR-TC#1 cells (Figure 2A and B). The amplicons on chromosome 4qC5 and C6 are made up of several short, closely spaced segments ranging from ∼5 to ∼50 copies. The 9qA1 amplicon, in contrast, consists of two adjacent segments with ∼18 and ∼22 copies (Figure 2B). These amplicons encode the oncogenes *c-Jun* (chr4qC5), *Yap1* (chr9qA1), *Birc2* and *Birc3* (both chr9qA1) as well as *Dcun1d5* (chr9qA1) and the leptin receptor (chr4C6). These findings suggest that the vGPCR-TC#1 cell line, but neither the vGPCR-TC#2 nor the parental vGPCR-3T3 cell line, is genomically unstable.

### vGPCR-TC#1 and vGPCR-TC#2 cells differ markedly for vGPCR-controlled genes

**Figure 3:**
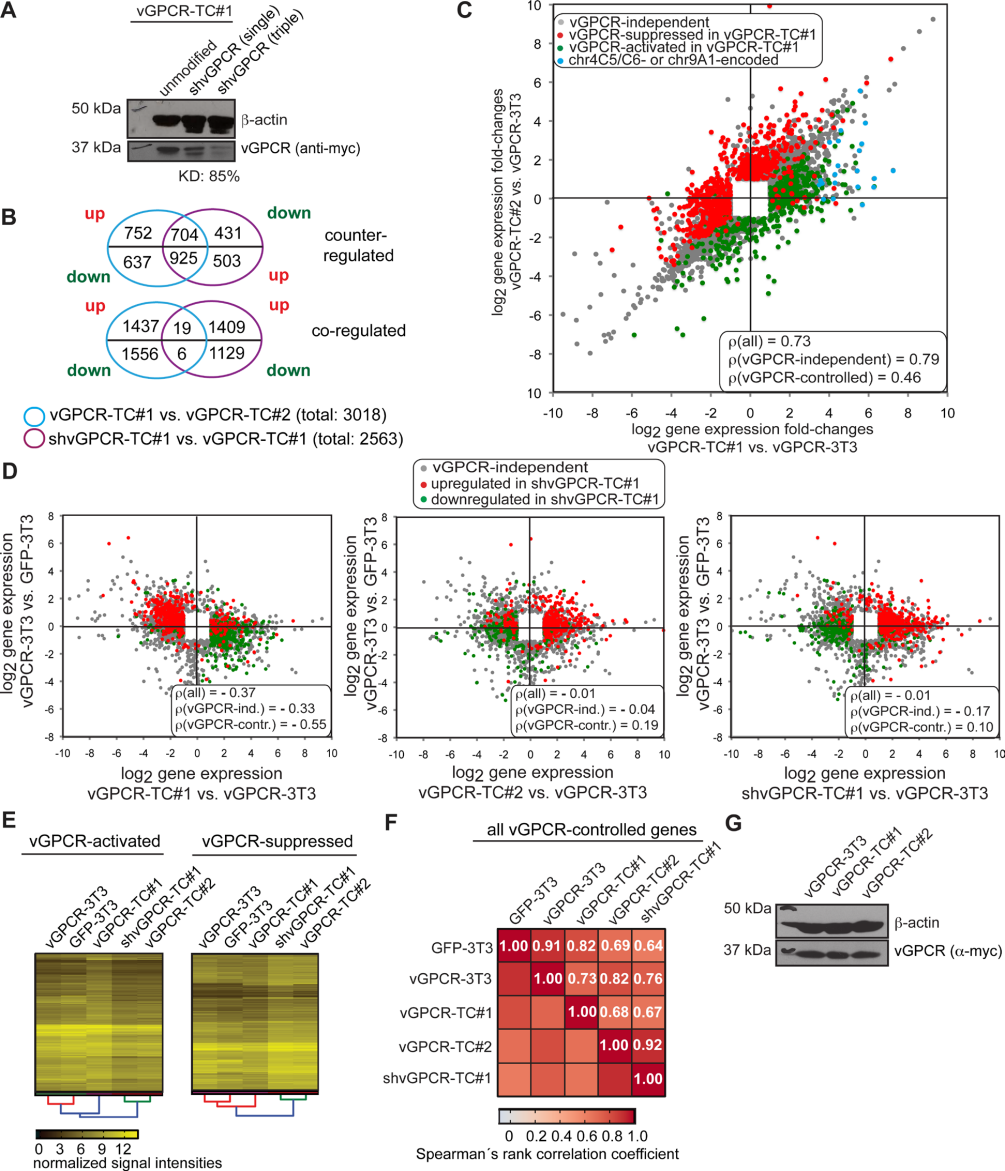
Identification of vGPCR-controlled genes in vGPCR-TC#1 cells by knockdown of the vGPCR **A.** Western blot analysis of the efficiency of vGPCR knockdown in vGPCR-TC#1 cells with either a single or three vGPCR-specific shRNAs: the simultaneous use of three shRNAs led to a knockdown efficiency (KD) of 85%. **B.** Venn diagrams showing the overlap between differentially expressed genes (fold-change ≥ 2, p ≤ 0.05) between vGPCR-TC#1 and vGPCR-TC#2 cells (blue circle) and vGPCR-controlled genes as identified by shRNA knockdown of the vGPCR (shvGPCR-TC#1 vs. vGPCR-TC#1, purple circle). The Venn diagrams break down this data to up- and downregulated genes for each comparison. The upper Venn diagram visualizes the overlap for genes that are counter-regulated, i.e. downregulated in vGPCR-TC#1 vs. vGPCR-TC#2 cells but upregulated upon knockdown of the vGPCR (vGPCR-suppressed genes) and *vice versa* for vGPCR-activated genes. The lower Venn diagram depicts the overlap for coregulated genes, e.g. downregulated in vGPCR-TC#1 cells and also downregulated in shvGPCR-TC#1 (vGPCR-activated) and *vice* versa for vGPCR-suppressed genes. **C.** Scatter plot comparing passaging-induced changes in gene expression for vGPCR-TC#1 and vGPCR-TC#2 cells, in each case relative to the parental cell line vGPCR-3T3. vGPCR-controlled genes upregulated upon vGPCR knockdown in shvGPCR-TC#1 cells (compared to vGPCR-TC#1 cells) are shown in red (vGPCR-suppressed) and genes downregulated upon vGPCR knockdown in green (vGPCR-activated); included are all probe sets that were significantly regulated in at least one comparison (fold-change ≥ 2, p ≤ 0.05); chr9A1 or chr4C5/C6-encoded genes are marked blue. The figure includes Spearman's rank correlation coefficients ρ for (i) all, (ii) vGPCR-independent, and (iii) and vGPCR-controlled gene probe sets. **D.** Scatter plots comparing passaging-induced gene expression changes (x-axis) versus vGPCR transformation-induced changes (y-axis) for vGPCR-TC#1 (left), vGPCR-TC#2 (middle) or shvGPCR-TC#1 cells (right); red and green dots mark vGPCR-controlled genes as described for panel C. **E.** Clustered heat maps comparing mRNA expression of vGPCR-controlled genes in GFP-3T3, vGPCR-3T3, vGPCR-TC#1, vGPCR-TC#2 and shvGPCR-TC#1 cells; left: vGPCR-activated genes, right: vGPCR-suppressed genes as identified by vGPCR knockdown in the vGPCR-TC#1 cell line. **F.** Correlation plot with color-coded Spearman's rank correlation coefficients ρ for vGPCR-controlled genes in indicated cell lines. **G.** Western blot of vGPCR abundances in vGPCR-3T3, vGPCR-TC#1 and vGPCR-TC#2 cells.

We have previously shown that the tumorigenicity of vGPCR-TC cell lines depends on the expression of the vGPCR oncogene as a stable knockdown of the receptor slowed down tumor growth in nude mice significantly [22]. Hence, we were interested in identifying genes that are controlled by the vGPCR and their possible contribution to the genomic instability and aggressive growth behavior of vGPCR-TC#1 cells. To this end, we knocked down vGPCR expression in vGPCR-TC#1 cells by lentiviral transduction with a set of three specific shRNAs. The knockdown efficiency in the newly generated shvGPCR-TC#1 cell line reached about 85 % on the protein level (Figure 3A). A comparison of the gene expression profiles of shvGPCR-TC#1 and vGPCR-TC#1 cells identified 1998 genes (2563 probe sets) to be controlled by the oncogenic receptor in vGPCR-TC#1 cells (Figure 3B, purple circles). The majority of these probe sets (1654 out of 2563, sum of intersections of upper and lower Venn diagram, Figure 3B) were also differentially expressed between the tumor cell lines vGPCR-TC#1 and vGPCR-TC#2 (Figure 3B). vGPCR-suppressed genes (upregulated in shvGPCR-TC#1 when compared to vGPCR-TC#1 cells) were enriched among genes that were downregulated in vGPCR-TC#1 cells relative to vGPCR-TC#2 cells while vGPCR-activated genes were predominantly higher expressed in vGPCR-TC#1 than TC#2 cells (upper Venn diagram, Figure 3B). The differential impact of the vGPCR on gene expression in vGPCR-TC#1 and vGPCR-TC#2 cells can be easily visualized by color coding vGPCR-controlled genes in a scatter plot that compares passaging-induced changes in gene expression in both tumor cell lines (Figure 3C). Evidently, almost all vGPCR-activated genes are higher expressed in the cell line vGPCR-TC#1 than #2 (green dots in Figure 3C) while vGPCR-suppressed genes are generally lower expressed in vGPCR-TC#1 than in vGPCR-TC#2 cells (red dots, Figure 3C).

Further insight into the remarkably different regulation of vGPCR-controlled genes in vGPCR-TC#1 and -TC#2 cells was obtained by color-coding of vGPCR-controlled genes in scatter plots that display passaging-induced (x-axis) versus vGPCR-transformation-induced (y-axis) changes in gene expression (Figure 3D). The vGPCR is, for example, largely responsible for the negative correlation between transformation- and passaging induced changes in vGPCR-TC#1 cells (Spearman's rank correlation coefficient ρ for vGPCR-controlled genes = −0.55; Figure 3D, left plot). This implies that changes in gene expression upon introduction of the vGPCR in BALB/c-3T3 cells were mostly reversed in vGPCR-TC#1 cells. The knockdown of the vGPCR in shvGPCR-TC#1 cells, however, adjusted the expression levels of vGPCR-controlled genes to the ones of vGPCR-TC#2 cells and abolished the negative correlation observed in vGPCR-TC#1 cells (ρ(all) = −0.01, for shvGPCR-TC#1 cells vs. ρ(_all)_ = −0.37 for vGPCR-TC#1 cells, Figure 3D left and right plot). The strikingly similar expression of vGPCR-controlled genes in shvGPCR-TC#1 and vGPCR-TC#2 cells could be confirmed by hierarchical cluster analysis (Figure 3E): vGPCR-controlled genes show similar expression levels in shvGPCR-TC#1 and vGPCR-TC#2 cells (ρ = 0.92, Figure 3F) that differ strongly from those in vGPCR-TC#1 cells (ρ = 0.67 and ρ = 0.68, respectively, Figure 3F). The vGPCR knockdown rendered gene expression in shvGPCR-TC#1 cells also more similar to their parental cell line vGPCR-3T3 (ρ = 0.76 vs. ρ = 0.73, Figure 3F). The differential expression of vGPCR-controlled genes is, however, not caused by a different abundance of the vGPCR in the tumor cell lines or the parental vGPCR-3T3 cell line (Figure 3G). Our results suggest that the marked difference in vGPCR-controlled gene expression between vGPCR-TC#1 and vGPCR-TC#2 cells is due to changes downstream of the vGPCR oncogene.

### vGPCR-dependent suppression of genome maintenance pathways in vGPCR-TC#1 cells involves downregulation of putative miRNA-34a target genes

**Figure 4:**
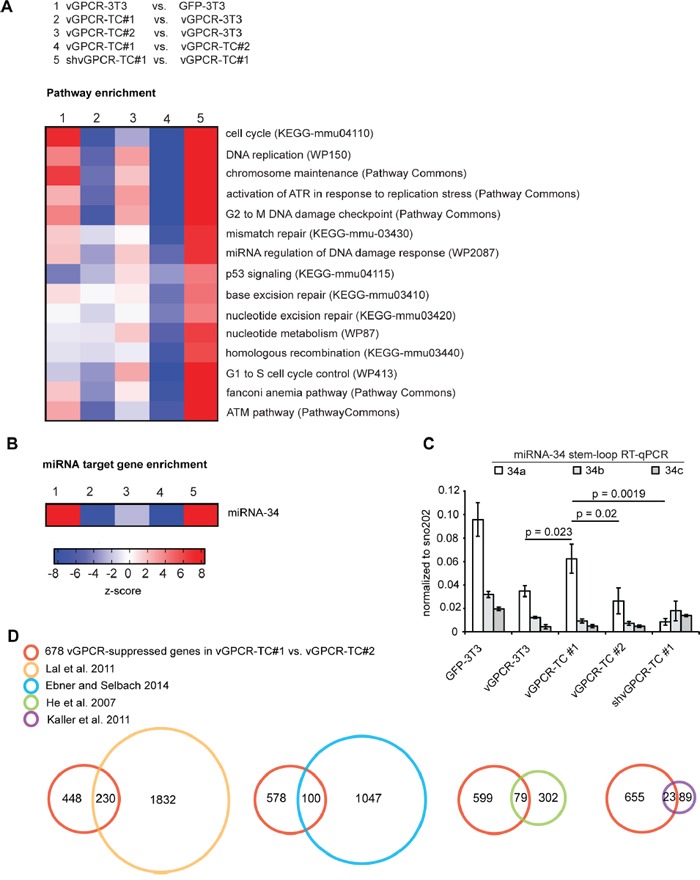
vGPCR- and miR-34a-dependent suppression of genome maintenance pathways **A.** Pathway enrichment analysis among differentially expressed genes (fold-change ≥ 2, p ≤ 0.05) for the indicated cell line comparisons; pathways enriched among upregulated genes show up in red and pathways enriched among downregulated genes show up in in blue according to the z-scores. **B.** Enrichment of miR-34 target genes (as identified by He *et al*. [9]) among up- or downregulated genes in the indicated comparisons. **C.** Expression of miR-34 family members (5p) as determined by stem-loop RT-qPCR. MicroRNA expression was normalized to the small RNA sno202; p-values have been determined by an unpaired t-test (n = 3). **D.** Identification of potential miR-34a targets among the 678 genes that are downregulated in vGPCR-TC#1 vs. vGPCR-TC#2 cells and upregulated upon knockdown of the vGPCR in shvGPCR-TC#1 cells (red circle). The Venn diagrams show the overlap of this gene set with experimentally identified miR-34a target gene sets taken from He *et al*. (orange) [9], Kaller *et al*. (purple) [26], Ebner and Selbach (blue) [27] and Lal *et al*. (green) [25].

The amplification and consequent overexpression of growth promoting genes encoded on chromosomes 9 (9qA1) and 4 (4qC5, 4qC6) most likely contributes to the higher tumorigenicity of vGPCR-TC#1 cells (Figure 1B). This raises the question about the underlying mechanisms that promote genomic instability and the possible role of the vGPCR in this process. To address these questions, we performed a pathway enrichment analysis that revealed a strong overrepresentation of pathways crucial for the maintenance of genomic integrity (Figure 4A) such as *DNA replication, Cell cycle, Chromosome maintenance, p53 signaling, G2 to M DNA damage checkpoint*, and *Activation of ATR in response to replication stress.* We further identified DNA repair pathways including *Homologous recombination, Base excision repair, Nucleotide excision repair, Mismatch repair* and the *Fanconi anemia pathway.* Genes from these pathways were highly enriched among genes that were downregulated in vGPCR-TC#1 cells when compared to vGPCR-TC#2 cells (Figure 4A, column 4) or the parental vGPCR-3T3 cells (Figure 4A, column 2), but not in vGPCR-TC#2 cells compared to the parental cell line (Figure 4A, column 3). Strikingly, these genes were largely suppressed by the vGPCR as the knockdown of vGPCR in shvGPCR-TC#1 cells restores their expression levels (Figure 4A, column 5).

The overrepresentation of genes of the pathway *miRNA regulation of DNA damage response* hinted at a possible involvement of miRNAs in the onset of vGPCR-induced genomic instability. A gene set enrichment analysis for microRNA target genes revealed a strong enrichment of microRNA-34 targets among genes downregulated in vGPCR-TC#1 cells relative to vGPCR-TC#2 or vGPCR-3T3 cells (Figure 4B, columns 2 & 4). As seen for the genome maintenance pathways, the expression of microRNA-34 target genes was also restored upon vGPCR knockdown (Figure 4B, column 5). This implies that at least one member of the microRNA-34 family is critically involved in the vGPCR-dependent suppression of genome maintenance mechanisms.

To assess the expression of microRNA-34 family members in our cell lines we used a stem-loop RT-qPCR approach [23, 24] specific for the mature form (5p) of miR-34a, b or c (Figure 4C). *miR-34a* was significantly higher expressed in both vGPCR-TC#1 and GFP-3T3 cells than in vGPCR-3T3 or the vGPCR-TC#2 cells. Interestingly, *miR-34a* expression is abolished in shvGPCR-TC#1 cells, indicating that the vGPCR activates *miR-34a* expression in vGPCR-TC#1 cells. In contrast, we did not observe a marked difference in *miR-34b* and *miR-34c* expression between the cell lines analyzed. The expression pattern of *miR-34a* fits the enrichment pattern of microRNA-34 target genes as depicted in Figure 4B: microRNA-34 targets are enriched among the downregulated genes if miR-34a-high cell lines (vGPCR-TC#1, GFP-3T3) were compared to miR-34a-low cell lines (vGPCR-3T3, vGPCR-TC#2 and shvGPCR-TC#1). Our results strongly suggest that miR-34a is indeed responsible for the enrichment pattern shown in Figure 4B.

Next, we assessed the number of potential miR-34a target genes within the set of the 678 vGPCR-suppressed genes (925 probe sets) that are downregulated in vGPCR-TC#1 vs. vGPCR-TC#2 cells (Figure 3B, upper Venn diagram). To this end, we overlaid our set of 678 vGPCR-suppressed genes with 4 data sets of experimentally identified miR-34a target genes (Figure 4D): He *et al.* [9] as well as Lal *et al.* [25] identified miR-34a targets on gene expression level using microarrays whereas Kaller *et al.* [26] and Ebner and Selbach [27] used a pulsed SILAC approach to identify targets at the protein level. In a combined overlay 284 out of 678 vGPCR-suppressed genes (42 %) were identified as experimentally validated miR-34a target genes. Another 125 of these 678 genes were bioinformatically predicted miR-34a target genes that were identified using TargetScan, RNAhybrid, miRanda, miRBase or Pictar.

### Tough Decoy RNA-mediated knockdown of miR-34a in vGPCR-TC#1 cells results in the upregulation of genome maintenance genes

**Figure 5:**
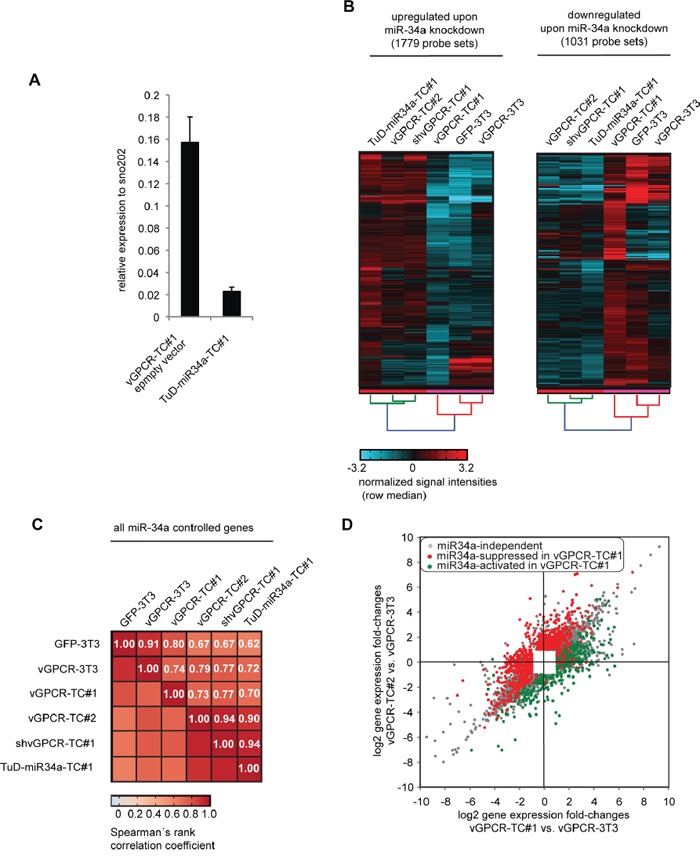
Identification of miR-34a-regulated genes in vGPCR-TC#1 cells by using a Tough Decoy RNA-mediated knockdown approach **A.** Efficiency of miR-34a knockdown in TuD-miR-34a-TC#1 cells that were generated by lentiviral transduction of vGPCR-TC#1 cells with a Tough Decoy RNA against miR-34a-5p. miR-34a-5p expression has been assessed by stem-loop RT-qPCR and normalized to sno202. Knockdown efficiency was determined via the ratio of TuD-miR-34a-TC#1 to vGPCR-TC#1 pLKO.1 neo (empty vector control). **B.** Cluster analysis for genes that were significantly regulated by the knockdown of miR-34a. The clustered heat map to the left compares the mRNA expression level for all probe sets that were upregulated following miR-34a knockdown in TuD-miR-34a-TC#1 vs. vGPCR-TC#1 cells, the heat map to the right all genes that were downregulated as a result of the miR-34a knockdown. **C.** Matrix with Spearman's rank correlation coefficients ρ of miR-34a-regulated genes (gene probe sets) as determined by the miR-34a knockdown for the indicated cell lines. **D.** Scatter plot comparing passaging-induced changes in gene expression for vGPCR-TC#1 and vGPCR-TC#2 cells, each relative to the parental cell line vGPCR-3T3. miR-34a-controlled genes that were upregulated upon miR-34a knockdown in TuD-miR34a-TC#1 cells (compared to vGPCR-TC#1 cells) are shown in red (miR-34a-suppresed) and those that were downregulated upon miR-34a knockdown are shown in green (miR-34a-activated); included are all probe sets that were significantly regulated in at least one comparison (fold-change ≥ 2, p ≤ 0.05).

So far, our data suggested that the vGPCR-mediated upregulation of miR-34a in vGPCR-TC#1 cells led to the suppression of the genome maintenance mechanisms. To verify this hypothesis, we knocked down miR-34a (5p) in vGPCR-TC#1 cells by a Tough Decoy RNA (TuD) approach [28] and analyzed the gene expression profile of the resulting TuD-miR-34a-TC#1 cell line. The knockdown of miR-34a (∼85%, Figure 5A) led to a significant change in the expression levels of 2164 genes (2810 probe sets; Figure 5B). Strikingly, we observed highly similar gene expression profiles for TuD-miR-34a-TC#1 cells and shvGPCR-TC#1 cells (ρ = 0.94, Figure 5C). Consequently, the distribution of miR-34a-regulated genes (Figure 5D) mimicked the one of vGPCR-regulated genes (Figure 3C) in a scatter plot depicting passaging-induced gene expression changes for vGPCR-TC#1 and -TC#2 cells. This confirms that miR-34a plays a major role downstream of the vGPCR oncogene. In total, we identified 707 genes that were suppressed by both the vGPCR- and miR-34a, many of which belong to genome maintenance pathways (Supplementary Table 2).

**Figure 6:**
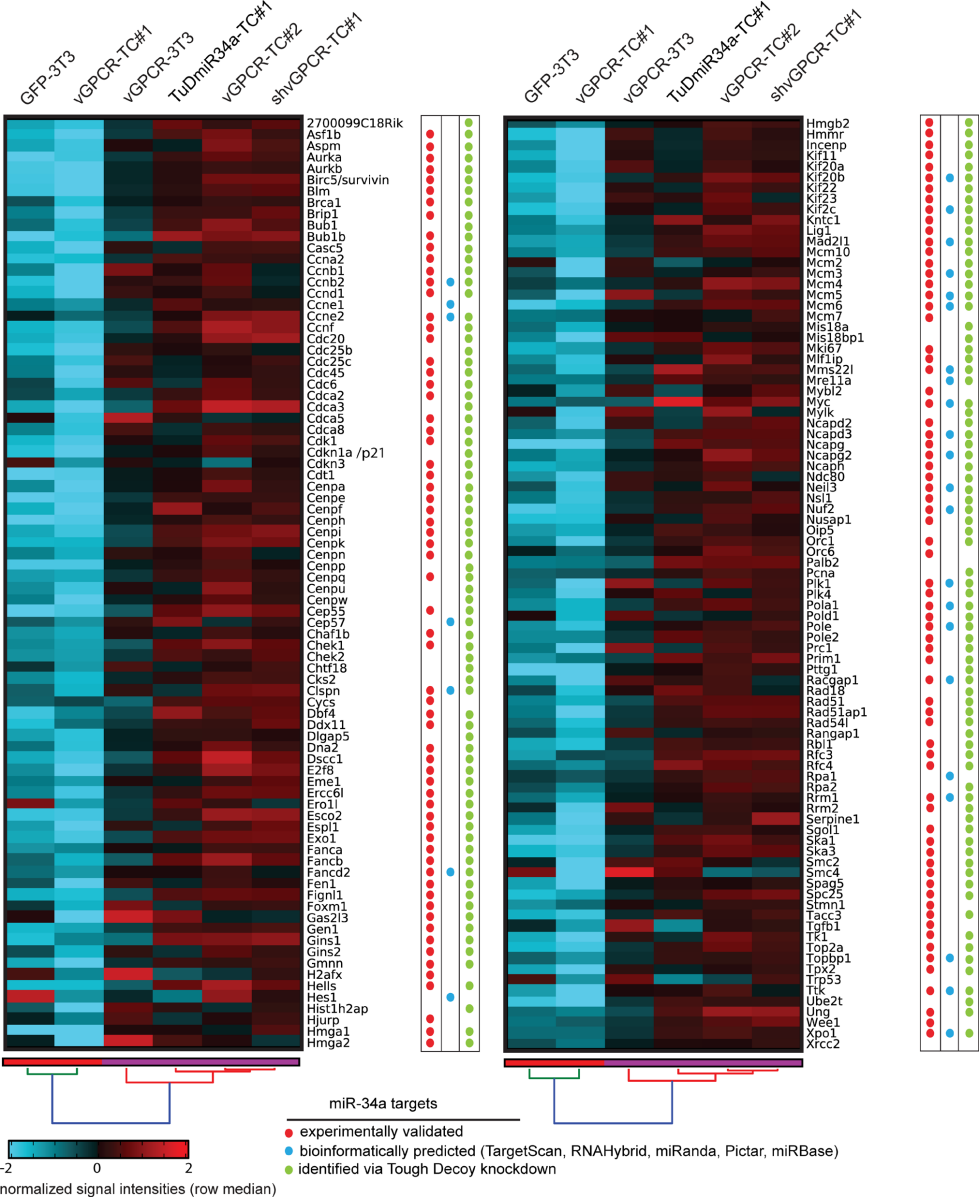
Genes from genome maintenance pathways are suppressed by both the vGPCR and miR-34a The clustered heat map depicts the signal intensities of vGPCR-suppressed genes from genome maintenance pathways that were significantly downregulated in vGPCR-TC#1 vs. vGPCR-TC#2 and upregulated in shvGPCR-TC#1 vs. vGPCR-TC#1 cells. miR-34a target genes are marked by (i) red dots for experimentally identified targets (references see column “Pubmed ID” in Supplementary Table 3); (ii) blue dots for bioinformatically predicted targets using TargetScan, Pictar, miRanda, miRBase, or RNAHybrid; and (iii) green dots for miR-34a target genes identified via Tough Decoy knockdown of miR-34a-5p.

The heat map in Figure 6 visualizes the expression levels of selected genes stemming predominantly from genome maintenance pathways. In Table 1, these genes have been classified according to their Gene Ontology (GO) terms showing that many genes are associated to the cell cycle, DNA repair, DNA replication or chromosome organization. Pathway associations taken from the KEGG pathways, WikiPathways, and Pathway Commons databases are listed in Supplementary Table 2 while Supplementary Table 3 contains the corresponding GO terms, fold-changes, bioinformatically predicted miR-34a target genes and references for experimentally identified miR-34a target genes. The color tags in Figure 6 mark miR34-a target genes as experimentally identified in other studies (red dots), bioinformatically predicted (blue dots) or identified by our Tough Decoy RNA-mediated knock down (green dots). The heat map clearly shows that the expression levels of the genome maintenance genes were much lower in cell lines expressing high levels of miR-34a (GFP-3T3 and vGPCR-TC#1) than in cell lines expressing low levels of miR-34a (vGPCR-3T3, vGPCR-TC#2, shvGPCR-TC#1, TuD-miR34a-TC#1). This result was confirmed for 10 randomly selected genes by RT-qPCR (see Supplementary Figure 1).

**Table 1:** Genes from genome maintenance pathways grouped by Gene Ontology (GO) terms

Gene Ontology term	Genes
Cell cycle	Aurka, Ccna2, Ccnb1, Ccnb2, Cnd1, Ccne1, Ccne2, Cdc20, Cdc25b, Cdc25c, Cdk1, Cdc45, Cdc6, Cdkn1a/p21, Cdkn3, Cep57, Cks2, Foxm1, Gas2l3, Kif20b, Mybl2, Plk1, Plk4, Rbl2, Rrm2, Spag5, Spc25, Tgfb1, Trp53, Wee1
Cell cycle arrest	Ccnd1, p21/Cdkn1a, Cdkn3, Chek1, Chek2, Gas2l3, Kif20b, Tgfb1, Trp53
CENP-A containing nucleosome assembly	Casc5, Cenpa, Cenpi, Cenpn, Cenpp, Cenpq, Cenpu, Cenpw, Hjurp, Mis18bp1
Centrosome	Aurka, Kif11, Cep55, Cep57, Aspm, Ccnf, Xpo1, Sgol1, Xrcc2
Checkpoints	Bub1, Bub1b, Mad2l1, Plk1, Ttk, Wee1, Brip1, Casc5, Cdc20, Cdc6, Chek1, Chek2, Clspn, Dbf4, H2afx
Chromosome condensation	Cdca5, Ncapd2, Ncapd3, Ncapg, Ncapg2, Ncaph, Smc2, Smc4
Chromosome segregation	Aurkb, Birc5, Bub1, Bub1b, Cdca2, Cenpe, Cenpf, Dlgap5, Ercc6l, Esco2, Espl1, Incenp, Kif2c, Kntc1, Mis18a, Ndc80, Nuf2, Pttg1, Rangap1, Sgol1, Ska1, Ska3, Spag5, Spc25
Mitotic spindle organization	Nusap1, Nsl1, Rangap1, Stmn1, Tacc3, Tpx2, Ttk
Cytokinesis/cell division/mitosis	Aurkb, Cep55, Cep57, E2f8, Gas2l3, Kif20a, Kif22, Kif23, Nusap1, Racgap1, Prc1, Xrcc2
DNA replication	Ccne1, Ccne2, Cdc25c, Cdc6, Cdk1, Cdc45, Cdca2, Cdca3, Cdt1, Chaf1b, Chek1, Chtf18, Clspn, Dbf4, Dna2, Dscc1, Fen1, Gins1, Gins2, Gmnn, Hmga1, Mcm10, Mcm2, Mcm3, Mcm4, Mcm5, Mcm6, Mcm7, Orc1, Orc6, Pcna, Pola1, Pold1, Pole, Pole2, Prim1, Rfc3, Rfc4, Rrm1, Rrm2, Tk1, Top2a, Rpa1, Rpa2, Xrcc2
DNA damage response	Chek1, Chek2, Topbp1, Clspn, Blm, Brca1, Rpa1, Rpa2, H2afx,
DNA repair	Blm, Brca1, Cdk1, Chaf1b, Clspn, Dna2, Eme1, Esco2, Exo1, Fanca, Fancb, Fancd2, Fen1, Fignl1, Foxm1, Gen1, H2afx, Hmga1, Hmga2, Hmgb2, Hmmr, Lig1, Mms22l, Mre11a, Neil3, Palb2, Pcna, Pola1, Pole, Pole2, Pttg1, Rad18, Rad51, Rad51ap1, Rad54l, Rfc3, Rfc4, Rpa1, Rpa2, Smc2, Smc4, Top2a, Topbp1, Trp53, Ube2t, Ung
DSB repair via homologous recombination	Blm, Brca1, Dna2, Eme1, Exo1, Fignl1, Gen1, H2afx, Mms22l, Palb2, Rad51, Rad51ap1, Rad54l, Rpa1, Rpa2, Xrcc2

In a second approach we analyzed the effect of miR-34a overexpression on global gene expression levels in vGPCR-3T3 and vGPCR-TC#2 cells. The lentivirally transduced cell lines miR-34a-vGPCR-3T3 and miR-34a-vGPCR-TC#2 showed a 9-fold and 3.4-fold increase in miR-34-a expression, respectively (Supplementary Figure 2A). Overexpression of miR-34a led to significant changes in the expression level of 2907 genes in vGPCR-3T3 cells but only 732 genes in vGPCR-TC#2 cells. Regarding the genome maintenance genes shown in Figure 6, we observed a downregulation of 51 genes in miR-34a-vGPCR-3T3 cells and 30 genes in miR-34a-vGPCR-TC#2 cells relative to the unmodified cell lines (see Supplementary Figure 2B).

Strikingly, almost all genome maintenance genes were upregulated upon the Tough Decoy knockdown while overexpression of miR-34a led to a downregulation of a subset of these genes. Taken together, these findings strengthen our argument that miR-34a is crucial for the repression of these genome maintenance genes in the setting of our mouse model for vGPCR-driven tumorigenesis and that it contributes to the onset of genomic instability.

## DISCUSSION

DNA tumor viruses have profound effects on host cell processes including those relevant to genome maintenance. The recognition of viral DNA by the host leads to an activation of DNA damage checkpoints and DNA repair mechanisms that protect genomic integrity and constitute a natural barrier to tumor progression. As part of their survival strategies DNA tumor viruses suppress the DNA damage response (DDR) to create an S-phase like environment suitable for viral replication [3, 4, 29, 30]. However, the suppression of genome maintenance mechanisms can induce genomic instability as reflected by the increased mutation rate and accelerated acquisition of chromosomal aberrations of the infected cells, which may lead to the onset of tumorigenesis [31, 32]. There is now increasing evidence that both latent and lytic KSHV proteins are able to induce genomic instability, however, a role of the KSHV-vGPCR in this process has so far not been described.

Until now, neither KSHV-vGPCR nor microRNA-34a has been implicated in the onset of genomic instability. However, using a mouse model of vGPCR-driven tumorigenesis, we clearly observed a vGPCR-dependent downregulation of numerous genome maintenance pathways and a striking accumulation of genetic aberrations in an aggressively growing tumor cell line expressing high levels of miR-34a. In stark contrast, a second tumor cell line expressing low levels of miR-34a showed no suppression of genome maintenance pathways and lacked massive genomic aberrations. By separately knocking down the vGPCR and miR-34a we could demonstrate that the downregulation of genome maintenance genes relied on the vGPCR and its downstream effector miR-34a. In addition, overexpression of miR-34a in either the parental vGPCR-3T3 or vGPCR-TC#2 cells led to a downregulation of a number of genome maintenance genes (see Supplementary Figure 2B), an effect that was stronger in vGPCR-3T3 than in vGPCR-TC#2 cells. This might reflect the higher expression of miR-34a in miR-34a-vGPCR-3T3 compared to miR-34a-vGPCR-TC#2 cells and most likely cell type-dependent effects of miR-34a expression.

Numerous miR-34a target genes identified in this study are crucially involved in processes such as DNA replication, chromosome maintenance, the DNA damage response and several DNA repair pathways including DNA double-strand break (DSB) repair via homologous recombination. In line with our results, Ebner and Selbach [27] and Lal and colleagues [25] have recently shown an enrichment of certain genome maintenance pathways among miR-34a target genes: DNA replication and cell cycle in the case of Ebner and Selbach and DNA replication, cell cycle, G1 to S control and DNA repair in the case of Lal *et al.* [25]. A study by Kofman *et al*. [33] demonstrated that miR-34a can induce DNA damage by greatly increasing the number of cells that undergo mitotic catastrophe; as this induced apoptosis, the authors concluded that miR-34a contributes to genome surveillance. A survival of only a few of these genomically unstable cells could, however, mark the onset of cancer.

Another crucial aspect for the maintenance of genomic integrity is accurate DNA replication [34]. We found that miR-34a targets many factors involved in the initiation of DNA replication, such as most components of the CMG (Cdc45, MCM, GINS) helicase, e.g. Cdc45, Mcm2-7, Gins1, Gins2 as well as Cdt1, Cdc6, and Mcm10. Downregulating these factors diminishes the cellular capacity to deal with replication stress, as marked by stalled or collapsed replication forks [34, 35], by reducing the overall number of licensed origins. An excess of licensed, but dormant, origins that are only activated upon replication stress is necessary to ensure complete replication of the genome [36]. In this sense, an excess of Mcm proteins prevents genomic instability [37, 38], whereas insufficient levels of Mcm can cause genomic instability [39, 40] and cancer [41]. Furthermore, many DNA repair pathways including base excision repair (BER), nucleotide excision repair (NER) and mismatch repair (MMR) as well as the DSB repair via HR are suppressed in a vGPCR- and miR-34a-dependent manner. The tumor suppressor BRCA1, for example, is critically involved in DSB repair, favoring the failsafe HR over the more error-prone NHEJ pathway [42]. BRCA1 also possesses an HR-independent function in the repair of DNA interstrand crosslinks (ICL) as it unloads the CMG complex from stalled replication forks [43]. It has recently been described as a miR-34a target [9, 25], which we verified by the Tough Decoy knockdown of miR-34a (see Figure 6).

Other target genes of miR-34a comprise Clspn, Topbp1 or Blm. Claspin (Clspn) is a mediator in the DNA damage response and important stabilizer of replication forks [44] and its depletion increases the formation of chromosomal aberrations. Topbp1, another mediator in the DDR, interacts with the RecQ helicase BLM to maintain genomic stability [45]. BLM suppresses the recombination of sister strands during the resolution of the double Holliday junction in HR thereby preventing copy number changes [46]. Disruption of the *BLM* gene causes Bloom's syndrome, a disease that is marked by a strong predisposition to genomic instability and cancer [47]. Both the vGPCR and miR-34a suppress also a large number of genes involved in chromosome condensation (e.g. Smc2, Smc4, Ncapd2, Ncapd3, Ncapg), chromosome segregation (e.g. Bub1, Esco2, Espl1), organization of the centrosome (e.g. Cep55, Cep57) and the establishment of the mitotic spindle (e.g. Nusap1, Nsl1). They also suppress most members of the chromosomal passenger complex, including Aurora kinase B, Incenp and survivin, being the central regulator of mitosis. We also found a large number of cell cycle genes to be downregulated in the miR-34a-high tumor cell line including the well-known miR-34a target gene Ccne2 [9, 25, 27] and many genes involved in the induction of cell cycle arrest such as p21/Cdkn1a or Gas2l3 whose depletion has been shown to induce genomic instability [48].

Until now, miR-34a has been mostly assigned a proapoptotic, tumor-suppressive function [9, 12, 49]. However, the effect of endogenous or artificial miR-34a expression seems to be highly cell type- and context-dependent: For example, a knockdown of miR-34a in HeLa cells that express this microRNA endogenously reduces their proliferation rate [19]. Similarly, miR-34a inhibits apoptosis and thus promotes the expansion of myeloid-derived suppressor cells [50]. Studies by Bhatt *et al*. [16] and Kato *et al*. [17] showed that miR-34a can act cytoprotectively by increasing the survival of cisplatin-treated and irradiated cells. In line with a potentially protumorigenic role, upregulation of miR-34a has been reported in many cancers [18-20, 51]. Although p53 is a well-known inducer of the microRNA-34 family [9, 12], Concepcion and colleagues [15] convincingly showed that microRNA-34 is not functionally required for p53-dependent processes in replicative senescence, oncogene-activation or the response to DNA damage. Moreover, miR-34a/b/c triple knockout mice showed no increased tumor formation rate, very much unlike p53 knockout mice, and miR-34-deficient thymocytes were equally resistant to radiation-induced apoptosis as their wild type counterparts.

The expression of miR-34a can also be induced independently of p53, e.g. by TAp73, retinoic acid phorbol ester or oncogenic stress [14] and NF-κB-binding to the miR-34a promoter has been shown to increase transcriptional activity [20]. Interestingly, Forte and colleagues [21] showed that *miR-34a* is highly expressed in KSHV-infected cell lines. Our data indicate that the vGPCR is critical for the upregulation of miR-34a in KSHV-infected cells, potentially also in an NF-κB-dependent manner since NF-κB is an important downstream signaling pathway of the vGPCR [52]. vGPCR-dependent upregulation of miR-34a could therefore aid virus replication and persistence by downregulating the DNA damage response and DNA repair. This hypothesis is supported by a study by Moody *et al.* [53] where the authors identified cellular target genes for the 12 KSHV-encoded microRNAs K1-K12. These include a number of genome maintenance genes such as MCM subunits, Plk1, Wee1, Bub1 or cyclins that are also downregulated by the vGPCR and miR-34a in our tumor model.

The similar abundance of the vGPCR in miR-34a-high and miR-34a-low tumor cell lines suggests that these cell lines differ in terms of vGPCR activity. As it is known that chemokines modulate the signaling activity of the vGPCR and that the modulation of vGPCR's signaling activity is crucial for its tumorigenic potential [54], we speculate that the differential vGPCR activity might result from an altered expression of vGPCR ligands. The vGPCR can bind several human and murine CXC chemokines that modulate its activity by either acting as agonists (e.g. CXCL1, CXCL3), antagonists (e.g. CXCL10, CXCL12) or neutral antagonists (e.g. CXCL5). The latter have no direct effect on receptor activity but compete with antagonists for receptor binding [55]. The generally low vGPCR activity in the BALB/c-3T3 background, as reflected by the moderate change in gene expression upon introduction of the vGPCR in BALB/c-3T3 cells could be a consequence of very high *Cxcl12* expression in all cell lines investigated in this study (Supplementary Figure 3). However, in the aggressively growing tumor cell line, receptor activity could be protected by the upregulation of the neutral ligand *Cxcl5* (32-fold relative to vGPCR-3T3) that competes with the antagonist CXCL12 for receptor binding. CXCL5 binding may enable a higher vGPCR activity and promote *miR-34a* expression whereas low vGPCR activity seems to suppress *miR-34a* expression as observed in the vGPCR-transformed BALB/c-3T3 cells or the less aggressive, miR-34a-low tumor cell line.

The genomic instability of the miR-34a-high tumor cell line was reflected in high-copy amplicons coding for the oncogenes *Yap1, Birc2, Birc3, Dcun1d5* (all on 9A1) or the leptin receptor (4C6). The chromosome 9A1 amplicon has been described before in mouse tumor models [56, 57] and corresponds to the human 11q22 amplicon that is frequently found in cancers, especially in squamous cell carcinomas of mucosal origin where it is associated with an aggressive phenotype and poor clinical outcome [58]. The 9A1 amplicon might be favorable for vGPCR-mediated tumorigenesis as it harbors oncogenes that are regulated by the vGPCR. Only recently, vGPCR-dependent Yap1 activation via suppression of the Hippo pathway has been shown [59]. Likewise, the *Birc3* gene is known to be vGPCR-controlled [60]. Hence, we observed a significant reduction in the expression of several chromosome 9A1-encoded genes upon vGPCR knockdown (Supplementary Figure 4), which points to a supportive role of this amplicon in vGPCR-dependent transformation and a possible function of these genes in Kaposi's sarcomagenesis. The leptin receptor, an important gene in the 4C6 locus, may be similarly important as it is known to induce proliferation and secretion of proangiogenic and immunosuppressive VEGFA [61]. We could also show that leptin activates potent oncogenic pathways depending on JAK2/STAT3, AKT and ERK1/2 in the tumor cell line that carried a high-copy amplicon on the locus of the leptin receptor (Supplementary Figure 5).

Summing up, our data provide compelling evidence that high miR-34a levels as induced by the KSHV-encoded oncogenic chemokine receptor vGPCR can promote genomic instability, an enabling hallmark of cancer and powerful toolkit for developing cancer cells, which boosts the mutation rate. In view of recent publications that also support a protumorigenic role of miR-34a, the concept of using miR-34a mimetics in cancer therapy [13, 14] should be viewed with caution as such a replacement therapy might promote genomic instability.

## MATERIALS AND METHODS

### Cell lines and cell culture

The generation of the BALB/c-3T3-derived cell lines vGPCR-3T3 and GFP-3T3 has been described previously [22]. The tumor-derived cell lines vGPCR-TC#1 and -#2 were established from tumors grown in BALB/c mice as described by Thirunarayanan *et al*. [22]. In brief, vGPCR-3T3 cells (5×10^5^ cells/mouse) gave rise to tumors in BALB/c nude mice. Fragments of these tumors were transplanted s.c into the flank of immunocompetent BALB/c mice. Tumors from BALB/c mice were then minced and tumor-derived cells cultured for two to three weeks under puromycin selection, resulting in a puromycin-resistant population of vGPCR expressing 3T3 tumor cells. shvGPCR-TC#1 and TuD-miR-34a-TC#1 cells were generated by lentiviral transduction of vGPCR-TC#1 cells with either vGPCR-specific shRNAs or a miR-34a-5p-specific Tough Decoy RNA (see below).

All cell lines were grown in low glucose DMEM supplemented with 10 % heat-inactivated newborn calf serum, penicillin/streptomycin, non-essential amino acids and L-glutamine; shvGPCR-TC#1 and TuD-miR-34a-TC#1 cells were maintained under 100 - 250 μg/ml G418 in the standard medium; HEK293T for lentivirus production were kept in high glucose DMEM, 10 % heat-inactivated fetal calf serum, penicillin/streptomycin, L-glutamine and sodium pyruvate.

### Lentiviral particle generation and transduction

#### Generation of shvGPCR-TC#1 cell line

vGPCR-specific shRNAs have been cloned in-between *Age*I and *Eco*RI restriction sites of pLKO.1 neo (a kind gift of Sheila Stewart). HEK293T cells have been co-transfected with shvGPCR-pLKO.1 neo, pLP1, pLP2 and pCMV-VSV-G (Invitrogen) using linear PEI (MW 40.000, Polysciences). Virus-containing supernatants were sterile-filtrated (0.45 μm) and concentrated by ultracentrifugation (3 hrs, 50.000 g, 4°C) or used freshly for transduction of vGPCR-TC#1 cells. Transduced cells were selected in medium containing 500 – 1000 μg/ml G418 (Genaxxon) and maintained in medium containing 100 – 250 μg/ml G418.

shRNA oligos (target sequence underlined):

shvGPCR#1-F: CCGGTCCTGGAATGAAACTCTAAA TACTCGAGTATTTAGAGTTTCATTCCAGGTTTTTG

shvGPCR#1-R: AGGACCTTACTTTGAGATTTATGAGC TCATAAATCTCAAAGTAAGGTCCAAAAACTTAA

shvGPCR#2-F: CCGGTGCTTGTGCAGACTTGAAAT TTCTCGAGAAATTTCAAGTCTGCACAAGCTTTTTG

shvGPCR#2-R: ACGAACACGTCTGAACTTTAAAGAG CTCTTTAAAGTTCAGACGTGTTCGAAAAACTTAA

shvGPCR#3-F: CCGGTACATCCGCTGCACTGTTAATT CTCGAGAATTAACAGTGCAGCGGATGTTTTTTG

shvGPCR#3-R: ATGTAGGCGACGTGACAATTAAGAG CTCTTAATTGTCACGTCGCCTACAAAAAACTTAA

#### Generation of TuD-miR-34a-TC#1 cell line

A Tough Decoy RNA (according to Haraguchi *et al*. [28] directed against the 5p product of mmu-miR-34a (miRBase accession number: MIMAT0000542) was cloned in-between the *Age*I and *Eco*RI restriction sites of pLKO.1 neo (a kind gift of Sheila Stewart). Lentiviral particle generation and transduction was performed as described above. The Tough Decoy RNA insert (TuD-miR-34a-5p) was synthesized by GeneArt, Lifetechnologies.

Sequence TuD-miR34a5p including *Age*I and *EcoR*I sites (italic)

*ACCGGT*GCTAGGATCATCAACACAACCAGC TAAATCTGACACTGCCACAAGTATTCTGGTCACA GAATACAACACAACCAGCTAAATCTGACACTGC CACAAGATGATCCTAGCACCGGATTTTTT*GAATTC*

#### Generation of miR-34a-overexpressing cell lines

vGPCR-3T3 and vGPCR-TC#2 cells were lentivirally transduced with MmiR3342-MR12 (GeneCopoeia) coding for mouse pre-miR34a. Transduced miR-34a-vGPCR-3T3 and miR-34a-vGPCR-TC#2 cells were selected using G418.

### Gene expression profiling and bioinformatic analyses

Total RNA was isolated using ZymoResearch's Quick-RNA™ MiniPrep kit. RNA quality was assessed with Agilent's Bioanalyzer 2100 using the RNA 6000 Nano kit. RNA was reverse-transcribed, labeled and fragmented using the Affymetrix 3′IVT Express Kit and hybridized to Affymetrix GeneChip 430 2.0 arrays. Cell lines have been analyzed in duplicates or triplicates. Affymetrix CEL files were analyzed using the open-source software AltAnalyze (Gladstone Institutes, University of California, San Francisco) using a moderated t-test. Differentially expressed genes were identified by a fold-change of at least 2 and a p-value (Benjamini-Hochberg adjusted) of ≤ 0.05. Scatter plots were generated in Microsoft Excel by plotting fold-changes (log_2_) in gene expression whereby probes had to be significantly regulated in at least one of the two comparisons shown in the plot. Spearman's rank correlation coefficients ρ were calculated using the following formula: r = 1 - ((6 *∑d2)/(n^2^ * (n-1)); n = number of elements in comparison, d = difference of ranks. Enriched pathways (from the KEGG, WikiPathway and PathwayCommon databases) as well as z-scores for microRNA-34 target genes and bioinformatically predicted miR-34a targets have been identified via GO Elite integrated into Altanalyze (data set for miRNA-34 targets was taken from He *et al*. [9] via the database Amadeus Metazoan compendium). Venn diagrams and clustered heat maps of gene expression data (log_2_ signal intensities) have been generated in AltAnalyze. Clustering was based on cosine similarity measures with (red-blue color scheme) or without (yellow-black color scheme) normalization on row median values. Raw data files were deposited in the ArrayExpress database (accession number E-MTAB-3393).

References (Pubmed IDs) for miR-34a target genes can be found in Supplementary Table 3.

### Relative protein quantification via stable isotope dimethyl labeling

Changes in relative protein abundance for the cell lines vGPCR-3T3, vGPCR-TC#1 and vGPCR-TC#2 were determined by dimethyl labeling. All pairwise comparisons were performed in triplicates. Protein extracts were prepared in denaturation buffer (6 M urea, 2 M thiourea, 10 mM HEPES-KOH pH8.0), sonicated to shear DNA and subsequently ultra-centrifuged at 100.000 g. Protein concentrations were determined by Bradford assay and 20 μg of each sample were taken for in-solution digestion. For tryptic in-solution digestion, samples were reduced with 1 mM tris(2-carboxyethyl) phosphine (TCEP) and free sulfhydryl groups carbamidomethylated using 5.5 mM choloroacetamide. Proteins were pre-digested with 0.5 μg sequencing grade endopeptidase LysC (Wako) for 3 hrs at room temperature and subsequently diluted with four volumes of 50 mM ammonium-bicarbonate. Tryptic digest was performed with 1 μg sequencing grade trypsin (Promega) for 8 h and stopped by acidification using 10% trifluoroacetic acid. The entire digest protocol was performed as described in Kanashova *et al*. [62]. Peptides were purified using C18 stage-tips (3M) [63]. The eluted peptides were lyophilized in a speed-vac and reconstituted with 100 μl 100 mM triethylammonium bicarbonate (TEAB) and dimethyl-labeled in solution [64] using light (+28 Da) or heavy formaldehyde (+32 Da) at a final concentration of 0.15 % in the presence of 0.8 % sodium cyanoborohydride. The reaction was carried out over night, quenched with 16 μl ABC buffer and acidified by adding 8 μl 50 % TFA. Dimethyl labeling was performed in an automated fashion on an xt PAL (CTC analysis) as described in Kanashova *et al*. [62]. Labeled samples were mixed in a 1:1 ratio and purified using C18 stage-tips. Samples were measured by LC-MS/MS on a Q Exactive Orbitrap mass spectrometer (Thermo Scientific) connected to a Proxeon nano-LC system (Thermo Scientific) in a data-dependent acquisition mode using the top 10 peaks for HCD fragmentation. 5 μl of sample was injected and a four-hour gradient (solvent A: 5 % acetonitrile, 0.1 % formic acid; solvent B: 80 % acetonitrile, 0.1 % formic acid) was applied eluting peptides at 4 % to 76 % acetonitrile using an in-house prepared nano-LC column (0.074 mm x 250 mm, 3 μm Reprosil C18, Dr. Maisch GmbH) at a flow rate of 0.25 μl/min. MS acquisition was performed at a resolution of 70.000 in the scan range from 300 to 1700 m/z. Dynamic exclusion was set to 30 s and the normalized collision energy was specified to 26. Data were analyzed with MaxQuant, version 1.3.0.5 [65], using a multiplicity of 2 for dimethylation analysis (modified epsilon amino groups of lysine plus modified N-terminal amino groups). Carbamidomethylation was set as a fixed modification while oxidized methionines and acetylated N-termini were set as variable modifications. An FDR of 0.01 was applied and Andromeda-based search was performed using a mouse Uniprot database (June 2012). Normalized ratios of the proteinGroups output were used to determine proteins with differential abundances. Significantly regulated proteins were defined to have a fold-change of at least 2.

### aCGH

Genomic DNA was extracted with Qiagen's DNeasy Blood & Tissue kit. Samples were digested overnight with proteinase K, DNA purified with phenol/chloroform/isoamylacohol pH 7.5-8 and labeled with Cy3; BALB/c reference DNA was labeled with Cy5. Labeling, fragmentation and hybridization to Agilent SurePrint G3, 4×180K arrays has been performed by Atlas Biolabs, Berlin, according to the manufacturer's instruction. GFP-3T3 and vGPCR-3T3 cells have been analyzed in triplicates, vGPCR-TC#1 cells in quintuplicates and vGPCR-TC#2 cells in duplicates. Data were evaluated using the software Genomics Suite (Partek) using the segmentation algorithm with the following parameters to identify all major copy number changes while avoiding oversegmentation: Minimum number of probes in a region ≥ 10, segmentation p-value ≤ 0.0001, signal to noise ratio 0.7, region parameter above ≥ 2.3 for amplifications, region parameter below ≤ 1.7 for deletions, region p-value ≤ 0.001. Raw aCGH data have been deposited in ArrayExpress (accession number E-MTAB-3394).

### *In vivo* tumor growth experiments

*In vivo* tumor growth experiments in mice were performed by EPO GmbH, Berlin-Buch, Germany. All mice were housed and cared for in accordance with state guidelines under an approved animal protocol. For each group, 8 male BALB/c mice aged 9-12 weeks were injected with 1*10^6^ tumor cells in 50 μl sterile PBS + 50 μl growth-factor reduced matrigel (BD Bioscience). Tumor growth was monitored regularly with a vernier caliper. Tumor volume was calculated using the modified ellipsoid formula.

### Western blotting

Cells have been lysed using M-PER reagent (Thermo Scientific); protein concentration was measured by a BCA protein assay (Thermo Scientific). Cellular lysates have been denatured in reducing SDS-loading buffer, separated by SDS-PAGE (Bio-Rad) and transferred to nitrocellulose membrane by wet blotting. vGPCR protein levels were assessed using an anti-myc antibody (Biolegend, #626802). Additionally used antibodies: leptin receptor (US Biological, #L1671-03R), phosho-Jak2 (#3776), phospho-Stat3 (#9145), phospho-Akt1 (#4060), phospho-Erk1/2 (#4377), all Cell Signaling Technology, β-actin (Abcam, #ab6276) and secondary HRP-conjugated antibodies from Jackson Immunoresearch. Western blots have been developed using ECL reagent (GE Healthcare).

### microRNA detection and quantification by stem-loop RT-qPCR

The small RNA fraction was isolated using Ambion's mirVana kit according to the manufacturer's instruction. RNA quality was determined using Agilent's Bioanalyzer 2100. RNA was reverse transcribed using a stem-loop primer specific for the mature form (5p) of mmu-miR-34a, b, c or sno202 as normalization control. Stem-loop primer have been designed according to Kramer [23], synthesized by BioTeZ (Berlin) and folded in a thermocycler using the following protocol: 10′ 95°C, followed by a temperature reduction of 2.5 K every 30 s until 80°C, 1 h at 75°C, 68°C, 65°C, 62°C and 60°C overnight (>10 h). qPCR was performed on an IQ5 thermocycler (Bio-Rad) using a universal reverse primer binding to the stem-loop region and a microRNA/sno202-specific forward primer; miRNA expression was normalized to the small RNA sno202 (Δc_T_ method) using a SYBR green/fluorescein master mix.

Primer sequences:

miR-34a-STL: GTCGTATCCAGTGCAGGGTCCGAG GTATTCGCACTGGATACGACACAACC

miR-34b-STL: GTCGTATCCAGTGCAGGGTCCGAGG TATTCGCACTGGATACGACACAATC

miR-34c-STL: GTCGTATCCAGTGCAGGGTCCGAGG TATTCGCACTGGATACGACGCAATC

sno202-STL: GTCGTATCCAGTGCAGGGTCCGAGGT ATTCGCACTGGATACGACCATCAG

Universal reverse primer (qPCR): CCAGTGCAGGGTC CGAGGTA

miR-34a-RT-F: GCAGTGTCTTAGCTGGTTG

miR-34b-RT-F: GGCAGTGTAATTAGCTGATT

miR-34c-RT-F: GCAGTGTAGTTAGCTGATTG

sno202-F: AGTACTTTTGAACCCTTTTCCA

### Realtime RT-qPCR

Total RNA was isolated using ZymoResearch's Quick-RNA™ MiniPrep kit and RNA quality controlled using Agilent's Bioanalyzer 2100. Up to 5 μg RNA was reverse transcribed with 10 μM OligodT_18_ primer (Thermo Scientific) and 5 Units Revert Aid H Minus reverse transcriptase (Thermo Scientific). The cDNA was quantified with a Nanodrop spectrometer (Thermo Scientific) and analyzed by realtime quantitative PCR using a SYBR green/fluorescin master mix on a BioRad IQ5 realtime thermocycler. Relative gene expression changes have been determined with the Δc_T_ method normalizing to β-actin. Errors have been calculated via the standard deviation SD of the c_T_ values: error bar = 2^-((c_T_*gene* - SD*gene*) – (c_T_*actin* + SD*actin*)) - 2^(c_T_*gene*-c_T_*actin*)

Realtime PCR primer:

Vegfa-F: CGGACGGGCCTCCGAAACCA

Vegfa-R: CAGCCTGGGACCACTTGGCAT

β-actin-F: GGCACCACACCTTCTACAATG

β-actin-R: GGGGTGTTGAAGGTCTCAAAC

#### Validation of chip data by realtime RT-qPCR

Ten randomly selected genes from Figure 6 have been analyzed by realtime qPCR. RNA was isolated using RNAisoPlus (Takara Bio, Japan). 1 μg RNA was transcribed using OligodT18 primer and PrimeScript reverse transcriptase (Takara Bio). qPCRs have been performed on Smart Cycler II (Cepheid, USA) using the 2X SYBR Premix Ex Taq II master mix (Takara Bio). Gene expression has been normalized to β-actin.

Primer sequences:

Brca1-F: CAGGCTTGACCCCCAAAGAAG

Brca1-R: ACCGGACCACCCATGAATAGC

Rad51-F: GCAGTAGCTGAGAGATACGGTC

Rad51-R: AATGCATTTGCCTGGCTGAAAGC

Aurkb-F: TTGCAGACTTTGGCTGGTCGG

Aurkb-R: GTCCACCTTGACAATCCGACG

Spag5-F: AAGCAAAGCATGAGAAACAGGCC

Spag5-R: GTTCTGGCACTTCATCTACTGC

Smc4-F: CTTCTATGTATCCAGAACCGCC

Smc4-R: GGCCTTTGGGTTTCATCATAGC

Mcm3-F: CCGTGGAGCTGGTTCAGTATG

Mcm3-R: AGTCTTTGGGGTGTGCACTTG

Mcm5-F: GGAGGCATTGAGACTGTTCCAG

Mcm5-R: AGACACCTGAGAGCCAATGGC

Ccnb1-F: AGGTGGAACTTGCTGAGCCTG

Ccnb1-R: GCACATCCAGATGTTTCCATCGG

Ska1-F: CATCTGCCTCAAGTGACGGC

Ska1-R: AGTCCAGGCTCCTCAGCTTTG

Bub1-F: CAGACAGGAATTCACAATGAGGC

Bub1-R: GACTCCTAGGAACACAGGCTG

### MTT proliferation assay

Cells were seeded in 96 well plates at a concentration of 15.000 cells/well. On the following day, cells were starved for 24 hrs in serum-free DMEM before adding 0, 50, 100 or 250 ng/ml leptin (PeproTech). 24 hrs after treatment, thiazolyl blue tetrazolium bromide (Sigma Aldrich) was added to a final concentration of 0.5 mg/ml. 4 hours later, medium was removed and cells lysed in 50 μl MTT solvent (4 mM HCl, 0.1 % Nonidet P-40 in isopropanol) and absorption at 570 nm measured with a microplate photoreader (BioTek Instruments, USA).

## SUPPLEMENTARY TABLES AND FIGURES









## Abbreviations

KSHV: Kaposi's sarcoma-associated herpesvirus
HHV-8: human herpesvirus 8
vGPCR: viral G protein coupled receptor
TuD: Tough Decoy RNA
miRNA: microRNA
TC: tumor cell line
KEGG: Kyoto Encyclopedia of Genes and Genomes
GO: Gene Ontology
GSEA: Gene Set Enrichment Analysis
KS: Kaposi's sarcoma
PEL: primary effusion lymphoma
MCD: multicentric Castleman's disease
DDR: DNA damage response
aCGH: array-based comparative genomic hybridization
DML: stable isotope dimethyl labeling
DSB: DNA double-strand break
HR: homologous recombination
NHEJ: non homologous end joining
NER: nucleotide excision repair
MMR: mismatch repair
BER: base excision repair.
