# Supplementary material for: MicroRNA-34a promotes genomic instability by a broad suppression of genome maintenance mechanisms downstream of the oncogene KSHV-vGPCR
Source: Oncotarget. 2016 Feb 8;7(9):10414–32. doi: 10.18632/oncotarget.7248 (PMC4891129; doi:10.18632/oncotarget.7248)
Supplement: Supplementary file 1 [file oncotarget-07-10414-s001.pdf]

SUPPLEMENTARY TABLES AND FIGURES

Supplementary Table S1: Stable isotope dimethyl labeling results. The table contains DML data for the cell line comparisons vGPCR-TC#1 vs. vGPCR-3T3 (Sheet 1), vGPCR-3T3 vs. vGPCR-TC#2 (Sheet 2) and vGPCR-TC#1 vs. vGPCR-TC#2 (Sheet 3) and a table summarizing the results of the DML analysis (Sheet 4)

See Supplementary File 1

Supplementary Table S2: Pathway annotations for genes suppressed by the vGPCR and miR-34a. This table lists all pathways from KEGG (Sheet 1), WikiPathways (Sheet 2) and Pathway Commons (Sheet 3) that are associated to the 707 vGPCR- and miR-34a-suppressed genes – comprising a large number of genome maintenance genes that are downregulated in both the shvGPCR-TC#1 and TuD-miR34a-TC#1 cell lines when compared to vGPCR-TC#1 cells. The pathways were identified using the software Altanalyze.

See Supplementary File 2

Supplementary Table S3: Fold-changes, Gene Ontology terms and miR-34a references for the genome maintenance genes. This table provides detailed information on genome maintenance genes suppressed by the vGPCR in vGPCR-TC#1 cells: gene probe sets, gene symbols, full gene names and selected gene ontologies, fold-changes in gene expression for selected comparisons and references (Pubmed IDs) for experimentally identified miR-34a target genes. Bioinformatically predicted miR-34a targets and genes identified as miR-34a targets in this study are marked accordingly in this table.

See Supplementary File 3

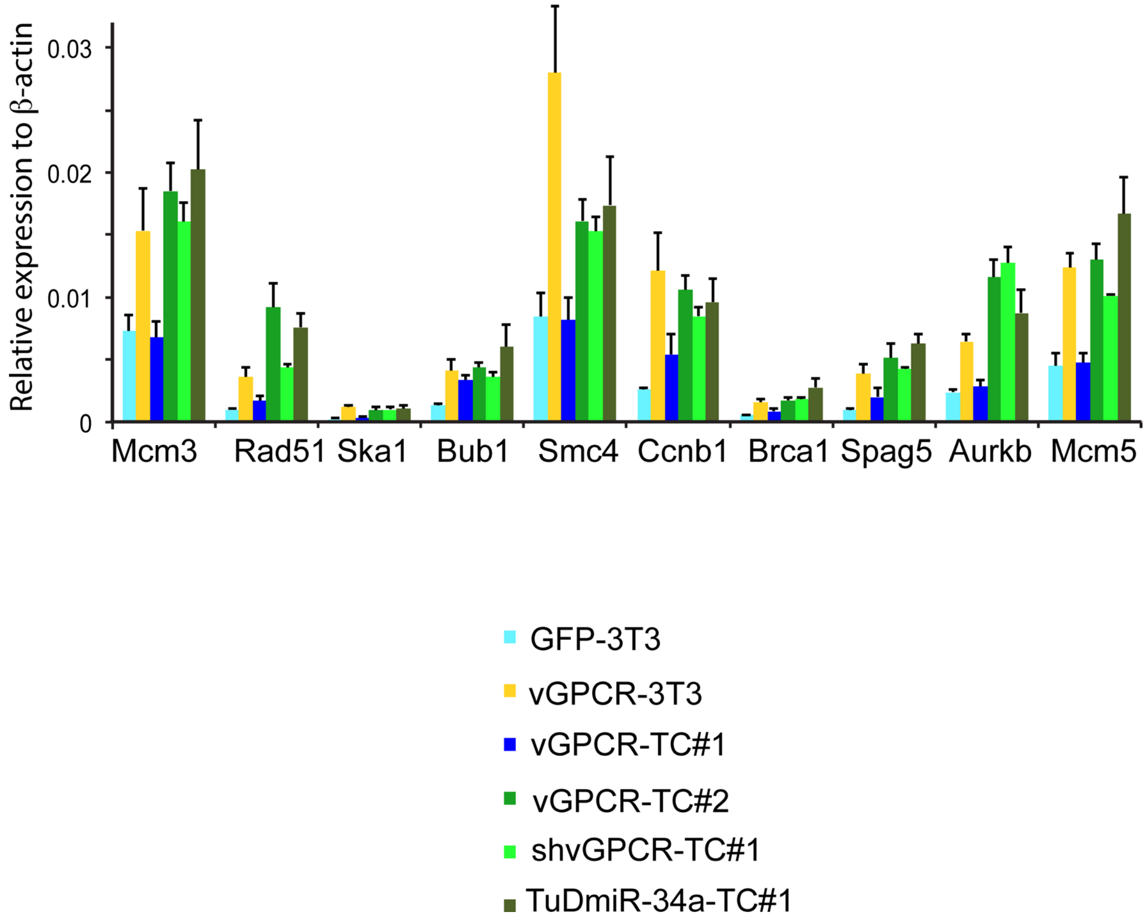

Supplementary Figure S1: Validation of microarray data by RT-qPCR. Ten randomly chosen, vGPCR- and miR-34a-suppressed genes stemming from the genome maintenance pathways have been analyzed by RT-qPCR to verify their gene expression levels (n≥3, mean+/-SD).

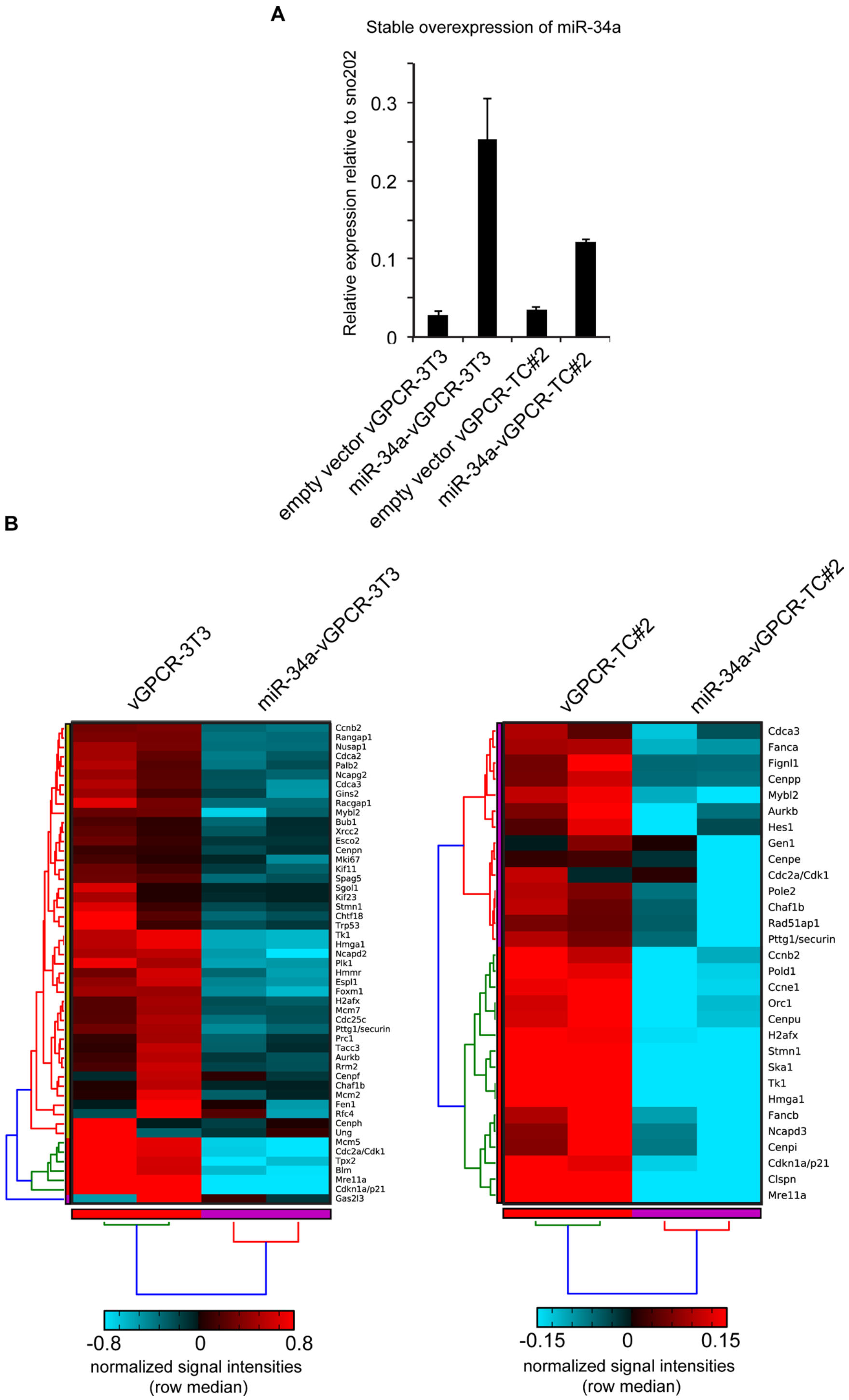

Supplementary Figure S2: Overexpression of miR-34a. A. Generation of the miR-34a overexpressing cell lines miR-34a-vGPCR-3T3 and miR-34a-vGPCR-TC#2. miR-34a levels were assessed by stem-loop RT-qPCR and normalized to sno202 expression. **B.** Downregulation of genome maintenance genes upon miR-34a overexpression in miR-34a-vGPCR-3T3 and miR-34a-vGPCR-TC#2 cells. The clustered heat maps show data from duplicate array analyses.

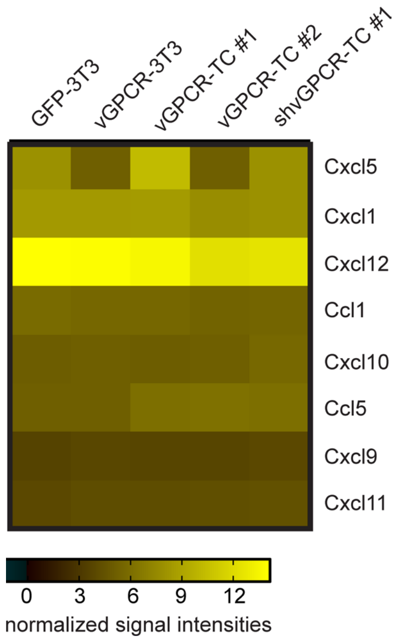

Supplementary Figure S3: Expression levels of vGPCR chemokine ligands. Heat map depicting the relative mRNA expression levels of all known vGPCR ligands present on Affymetrix GeneChip Mouse 430 2.0 arrays.

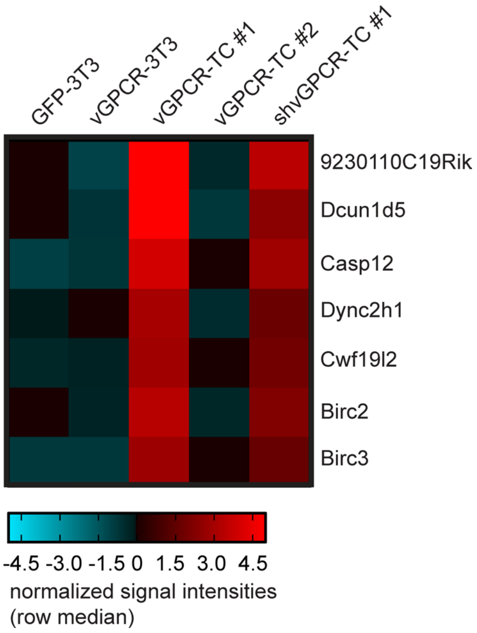

Supplementary Figure S4: vGPCR-controlled genes on chromosome 9A1. Clustered heat map of 9A1-encoded genes with reduced expression levels upon vGPCR knockdown (fold-change ≤ -2, p ≤ 0.05 in shvGPCR-TC#1 vs. vGPCR-TC#1 cells); signals were normalized to row medians.

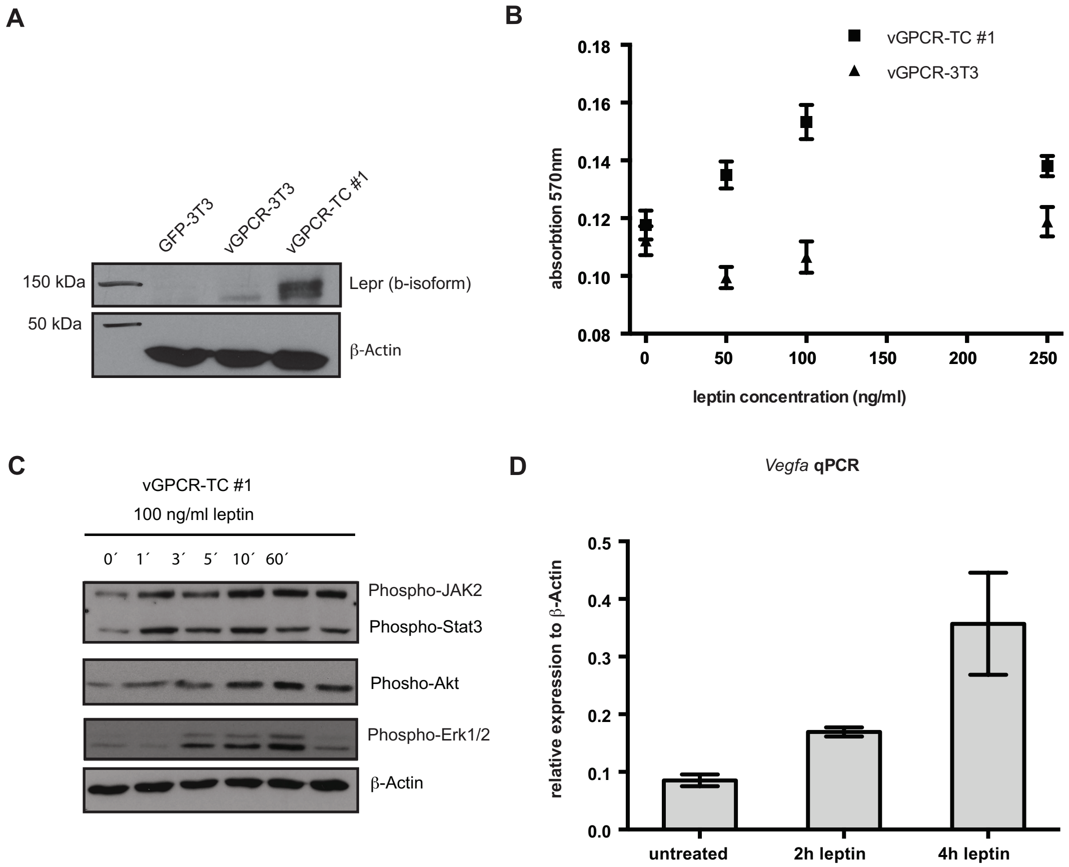

Supplementary Figure S5: Chromosome 4C6-encoded leptin receptor activates oncogenic signaling cascades, stimulates cellular proliferation and *Vegfa* expression in vGPCR-TC#1 cells. A. Expression levels of the full-length LepRB isoform. **B.** MTT proliferation assay for control and leptin-treated (24 hrs) vGPCR-3T3 and vGPCR-TC#1 cells. **C.** Activation of signaling cascades in vGPCR-TC#1 cells upon leptin treatment (100 ng/ml for the indicated time spans). **D.** Time-dependent induction of *Vegfa* gene expression in untreated and leptin-treated (100 ng/ml for 2 or 4h) vGPCR-TC#1 cells as assessed by realtime RT-qPCR.
